# Determining Surface Topography of a Dressed Grinding Wheel Using Bio-Inspired DNA-Based Computing

**DOI:** 10.3390/ma14081899

**Published:** 2021-04-11

**Authors:** Akihiko Kubo, Roberto Teti, AMM Sharif Ullah, Kenji Iwadate, Tiziana Segreto

**Affiliations:** 1 Division of Mechanical and Electrical Engineering, Kitami Institute of Technology, 165 Koen-cho, Kitami 090-8507, Japan; kuboak@mail.kitami-it.ac.jp (A.K.); ullah@mail.kitami-it.ac.jp (A.S.U.); iwadate@mail.kitami-it.ac.jp (K.I.); 2 Department of Chemical, Materials and Industrial Production Engineering, University of Naples Federico II, Piazzale Tecchio 80, 80125 Naples, Italy; tsegreto@unina.it

**Keywords:** image processing, surface topography, grinding, dressing, bio-inspired manufacturing, DNA-based computing

## Abstract

Grinding is commonly used for machining parts made of hard or brittle materials with the intent of ensuring a better surface finish. The material removal ability of a grinding wheel depends on whether the wheel surface is populated with a sufficiently high number of randomly distributed active abrasive grains. This condition is ensured by performing dressing operations at regular time intervals. The effectiveness of a dressing operation is determined by measuring the surface topography of the wheel (regions and their distributions on the grinding wheel work surface where the active abrasive grains reside). In many cases, image processing methods are employed to determine the surface topography. However, such procedures must be able to remove the regions where the abrasive grains do not reside while keeping, at the same time, the regions where the abrasive grains reside. Thus, special kinds of image processing techniques are needed to distinguish the non-grain regions from the grain regions, which requires a heavy computing load and long duration. As an alternative, in the framework of the “Biologicalisation in Manufacturing” paradigm, this study employs a bio-inspiration-based computing method known as DNA-based computing (DBC). It is shown that DBC can eliminate non-grain regions while keeping grain regions with significantly lower computational effort and time. On a surface of size 706.5 μm in the circumferential direction and 530 μm in the width direction, there are about 7000 potential regions where grains might reside, as the image processing results exhibit. After performing DBC, this number is reduced to about 300 (representing a realistic estimate). Thus, the outcomes of this study can help develop an intelligent image processing system to optimize dressing operations and thereby, grinding operations.

## 1. Introduction

Grinding is commonly used to machine workpieces made of hard or brittle materials with the intent of ensuring a better surface finish. In grinding, the shape of the grinding wheel and the randomly distributed abrasive grain trajectories are projected onto the workpiece surface [1]. Thus, the cutting-edge density of the abrasive grains, the number of protrusions of the grains, and the continuous cutting-edge intervals affect the abrasive grain trajectories and thereby, the surface finish [2,3,4]. The critical aspect is that the material removal ability of the abrasive grains deteriorates with time. Dressing and truing operations are performed at regular time intervals to restore the abrasive grains’ material removal ability. Various methods have been introduced so far to perform truing and dressing [5,6,7]. The effectiveness of truing and dressing operations is determined by quantifying wheel surface topography by identifying the regions and their distributions where the active abrasive grains reside.

Both analytical and experimental studies have been carried out to determine the surface topography of the grinding wheel. Numerous studies have been carried out to theoretically analyze the surface topography of the dressed grinding wheel and the properties of the finished surface [8,9,10]. Besides theoretical analysis, experimental and hybrid analyses have been a popular approach. In this regard, stylus-based measurements have largely been used. Matsui et al. [11] conducted a theoretical analysis and an experimental study of abrasive grain cutting-edge density for both the conical stylus and knife-edge stylus. One of the drawbacks of this approach is that the measurement results depend on the shape of the stylus. In addition to stylus-based measurements, non-contact measurements have also been used. Shoji et al. [12] measured the amount of protrusion of abrasive grains by stereo-photography and investigated the effect of this amount on grinding performance. Laser-based, non-contact measurements can be used to obtain the distribution of the grinding wheel outer surface heights with high accuracy [13,14,15]. Furthermore, the surface topography of the grinding wheel can be analyzed by various image processing methods [16,17,18,19,20,21,22,23,24]. In this case, the images of the grinding wheel work surface are prepared by pre-processing before the actual processing phase. These methods can measure a wide surface area quickly and quantify the grinding wheel surface conditions more realistically. For example, consider the work described in Hosokawa et al. [16]. In this article, the grinding wheel surface image was prepared in three steps: preprocessing of a color image, binary conversion, and main processing of a micro-chromatic image. The binary conversion was performed in the regions of abrasive grain and worn-flat areas with the aid of hue histograms, saturation histograms, and brilliance histograms. For example, consider some selected recent studies. Feng and Chen [17] showed that manual adjustment was required during the image processing of a grinding wheel surface, ensuring the right detection of the regions where the abrasive grains reside. Kawashita et al. [18] used an image processing system to determine a targeted abrasive grain’s wear due to the continuation of a grinding process. They extracted abrasive grain height information by processing the grinding wheel work surface using hue, saturation, and brilliance histograms. Abidi et al. [19] showed a digital image processing technique using the K-cluster iteration method to determine the abrasive and non-abrasive regions on a grinding wheel surface. Scanning electron microscope (SEM) images are also used for the sake of comparison. Kawashita et al. [20] developed an imaging technique wherein two light sources (ultraviolet and visible light sources) were used to take images of a grinding wheel surface with diamond abrasives. Kapłonek et al. [21,22,23] performed a series of studies showing the newly developed grinding wheel’s effectiveness by analyzing images of wheel surfaces and images of isolated abrasive grains. The original images were taken by scanning electron microscope (SEM) technology. Afterward, commercially available image processing systems were used to determine the topography of the grinding wheel surface. In topography analysis, the main challenge was to isolate the regions of grains, binders, and cavities in the binders [24]. The authors observed that cavities were also formed in the regions of grains.

However, one of the drawbacks of image processing-based topography analysis is that the underlying image processing method must remove the regions where the abrasive grains do not reside while maintaining at the same time the regions where the abrasive grains reside. Thus, innovative image processing techniques are needed to allow for effective distinction between non-grain regions and grain regions while simultaneously reducing the heavy computing load and time-consuming duration.

In the framework of the emerging paradigm of “Biologicalisation: Biological Transformation in Manufacturing” [24,25,26,27,28], the basic hypothesis is that future biologicalized manufacturing systems will develop along the three directions of (a) bio-inspiration, (b) bio-integration, and (c) bio-intelligence [28]. With reference to this assumption, the present paper aims to embrace the bio-inspiration manufacturing scheme defined as “Bio-inspired manufacturing is realized by transferring concepts concerning principles, functions, structures and/or solutions from the biosphere to the manufacturing technosphere” [28]. Within this concept, for overcoming the limits of current image processing-based topography analysis methods, this study presents a bio-inspiration based computing methodology, known as DNA-based computing (DBC) [29], with a focus on DBC’s effectiveness in helping remove non-grain regions while maintaining grain regions with a significantly reduced amount of computational effort and time.

After the introduction, this article is organized in the following sections: Section 2 presents the dressing conditions and pre-processing of the grinding wheel surface images. Section 3 describes the way DBC is performed on the pre-processed images to eliminate image regions that are not significant for surface topography determination. Section 4 presents the results of the DBC-driven image processing for grinding wheel surface topography determination. It also shows the relationship between dressing conditions and the distributions of active abrasive grains and discusses the implications of this study. Section 5 presents the concluding remarks of this research work.

## 2. Image Data Preparation

**Figure 1:**
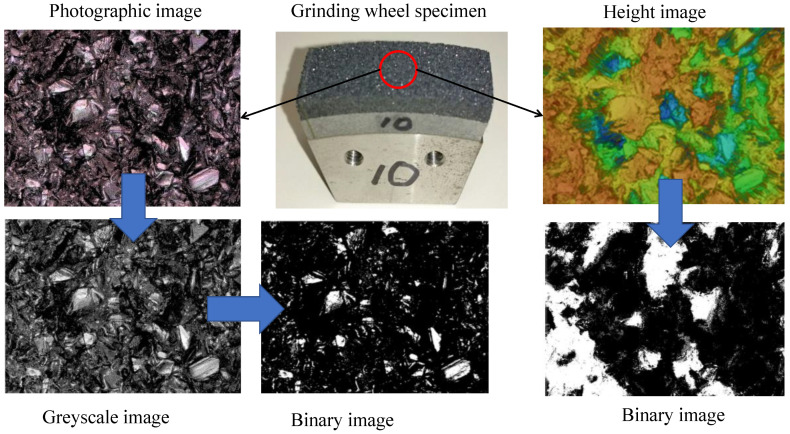
Image dataset preparation.

**Figure 2:**
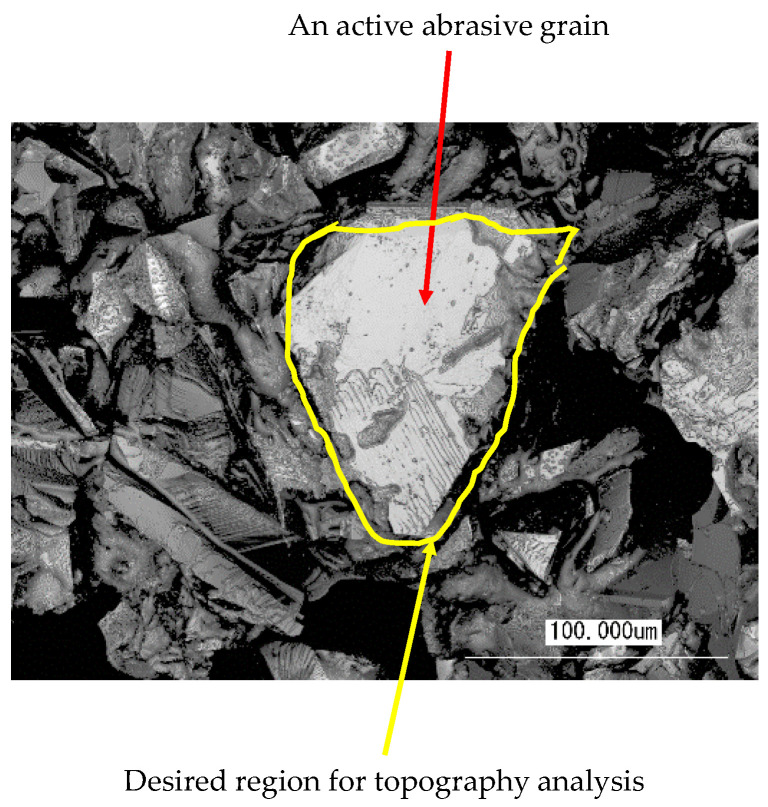
Image of an active abrasive grain and its vicinity.

Vitrified CBN grinding wheels (BN140M100V) were used for surface topography analysis. The grinding wheel, dresser specifications, dressing conditions (rotational speeds, velocity ratios, depth of cuts, and feed rates of dressing operations), and coolant type are listed in Table 1. The illustrative descriptions of dressing operations and relevant conditions can be found in [8,9,10]. As can be seen in Figure 1, the photographic images and surface height information of the grinding wheel surfaces under different dressing conditions are obtained using a short wavelength laser microscope (specification VK-9700, Keyence Corp., Osaka, Japan). The magnification factor was 20. For photographic imaging, the resolution of the data was 2048 pixels by 1536 pixels for surface size 706.5 μm in the circumferential direction and 530 μm in the width direction of the grinding wheel, respectively. Thus, unit pixel means here a 345 nm by 345 nm area. Figure 2 shows a typical image of the vicinity of an active abrasive grain. The white region marked as an active abrasive grain is the target area that must be distinguished from the other areas during the image processing-based topography determination process.

The color information of a pixel in a given image, denoted as *R* ∈ [0, 255], *G* ∈ [0, 255], *B* ∈ [0, 255], can be used to produce a grayscale image using the following formulae.

(1) $$Y=w_{R}R+w_{G}G+w_{B}B$$

(2) $$w_{R}+w_{G}+w_{B}=1$$

In Equations (1) and (2), *Y* ∈ [0, 255] is the numerical value of a pixel in the image, and the weights are *w_R_* = 0.2989, *w_G_* = 0.5870, and *w_B_* = 0.1140. A gray scale image can be converted into a binary image. In this case, a threshold *a* ∈ [0, 254] can be set to perform the following calculation for each pixel.

(3) $$Z=\{\begin{array}{ll} 1, & \mathrm{if}\;Y>a\\ 0, & \mathrm{otherwise} \end{array}$$

**Figure 3:**
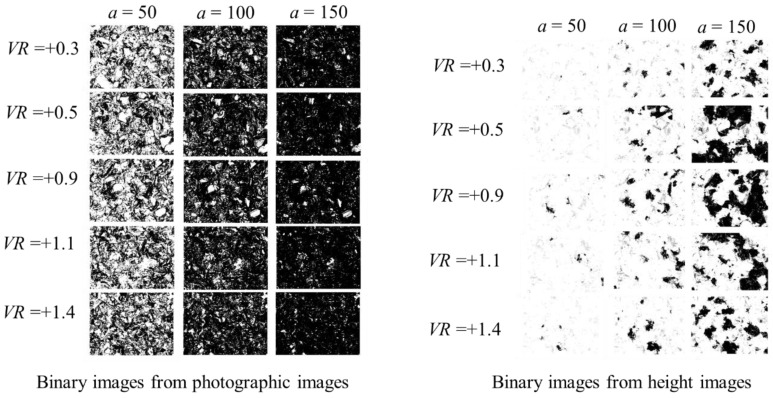
Comparison of binary images.

**Figure 4:**
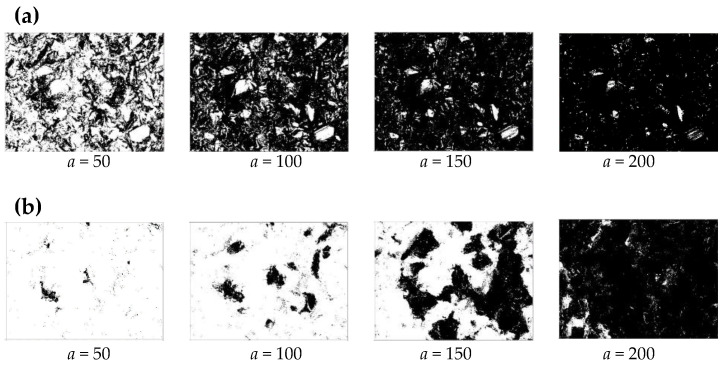
Significance of the threshold. (**a**) Photography-based binary image, (**b**) height-based binary image.

Thus, a pixel is represented by either 0 or 1 as *Z* ∈ {0, 1}. Since a binary image produced from a height image is less informative compared to the latter, as shown in Figure 3, the binary images created from photographic images are considered for further analysis. In addition, the threshold *a* is a critical parameter, as shown in Figure 3. The white regions (corresponding to 1 s) represent the regions where the abrasive grains might reside. From visual inspection, it is evident that the binary images extracted from the photographic images corresponding to *a* = 150 exhibit a realistic proportion of white and black areas. To be more specific, consider the case shown in Figure 4. This figure shows both photography- and height-based binary images for *VR* = +0.9 and *a* = 50, 100, 150, and 200, respectively. Other than *a* = 150, the white areas in the binary images do not correctly reflect the existence of the active abrasive grains. Accordingly, the binary images extracted from photographic images corresponding to *a* = 150 are utilized for grinding wheel surface topography analysis.

## 3. DNA-Based Computing (DBC)

**Figure 5:**
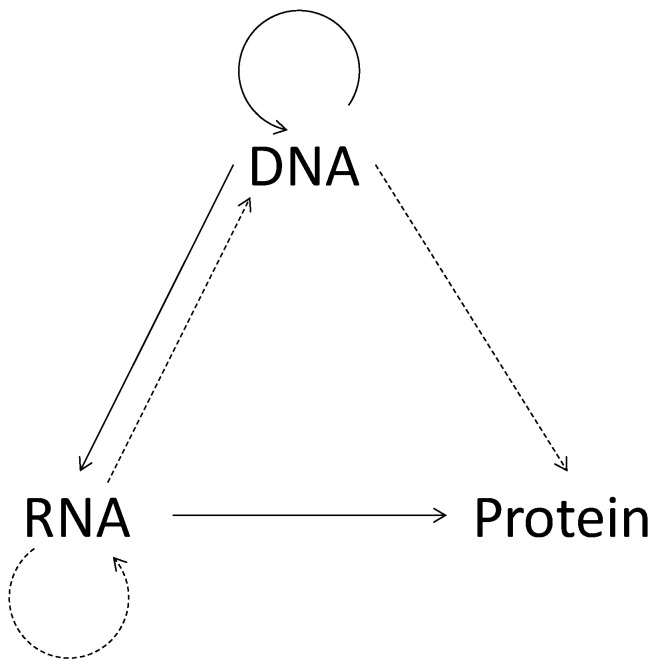
The central dogma of molecular biology [31].

Within the bio-inspiration scheme of “Biologicalisation in Manufacturing” [28], the bio-inspired DNA-based computing (DBC) method [29,30] is utilized according to the central dogma of molecular biology: Once the information of the DNA (sequence of four elements, A, C, G, and T) is passed to a protein (sequence of twenty elements (amino acid), it does not return [31]. This phenomenon is schematically illustrated in Figure 5. As a result, an information content increase takes place (see [32] for the details). Thus, DBC can help solve complex computational problems associated with intelligent manufacturing. It can help detect abnormalities in a manufacturing process even though there is a shortage of relevant data to make the decision [30]. It can identify similarities among tool wear images extracted from the same manufacturing process at different machining times [32,33]. It can help identify the similarity between chaotic time series of surface roughness, ensuring simulation process validity for intelligent machining. Furthermore, it can be used to construct digital twins [34]. Most importantly, it can identify complex boundaries in binary images for geometric modeling [29].

**Figure 6:**
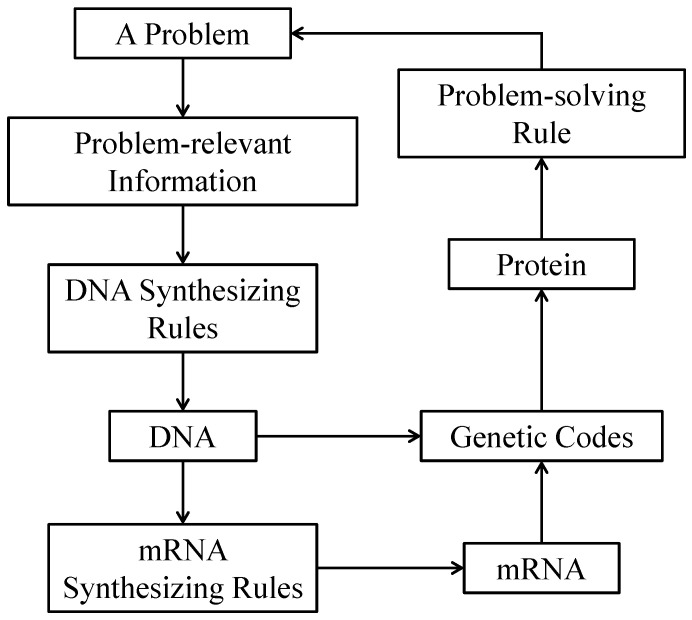
Schematic illustration of DNA-based computing (DBC).

The DBC method is schematically illustrated in Figure 6. As can be seen in the figure, DBC provides several decision rules for solving a given problem. To this purpose, first, the problem-relevant information is prepared. For this case, the problem-relevant information is a piece of a binary image extracted from a photographic image for *a* = 150. The problem-relevant information is transformed into DNA (a sequence or array of A, C, G, and T), as found in biological organisms. For this, user-defined DNA synthesizing rules are needed. The rules can be reset as many times as desired. Different DNA sequences or arrays can be produced by changing the problem-relevant information. The DNA sequences or arrays can be integrated to produce mRNA. Every three pairs of codes (denoted as codons) can be translated to form a protein (a sequence or array of amino acids). In this case, the universal genetic codes (see Table 2) that translate codons to corresponding amino acids are used.

**Figure 7:**
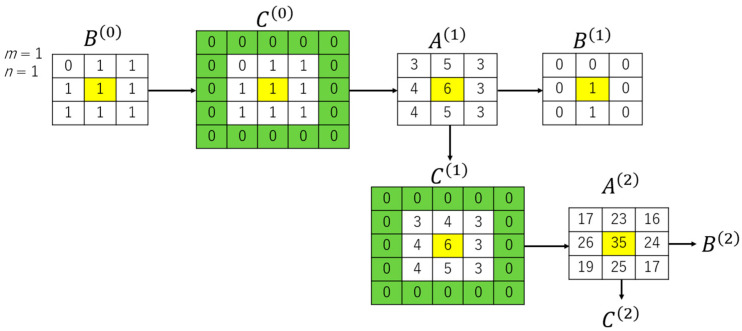
Pre-processing of binary images for DBC.

The formulations used to perform DBC on the binary images are described as follows: Let B^(0)^ be the binary image produced as described in Section 2. The binary image can be processed to produce arrays C^(0)^, adding padding as preferred. The padded array C^(0)^ can be transformed into A^(1)^, B^(1)^, and C^(1)^. The process continues, as schematically illustrated in Figure 7.

Thus, the following formulations hold:

(4) $$A_{k,l}^{\left(x+1\right)}=\sum_{i=-m}^{m}\sum_{j=-n}^{n}C_{k+i,l+j}^{\left(x\right)}$$

(5) $$B^{\left(x\right)}=\{\begin{array}{ll} 1, & A^{\left(x\right)}>b\\ 0, & \mathrm{otherwise} \end{array}$$

In Equation (5), *b*, *n*, *m*, *l*, and *k* are user-defined parameters. In this study, three arrays denoted as *B*^(1)^, *B*^(3)^, and *B*^(5)^ are produced for all *m* = *n* = 5. The modified binary arrays are compared with the original ones *B*^(0)^ to produce three different DNA arrays denoted as *D*^(1)^, *D*^(3)^, and *D*^(5)^. In doing so, different formulations can be employed. In this study, two formulations were used. One of the formulations was defined as the ACTG rule. The other was defined as the ACGT rule. The ACTG and ACGT rules, respectively, are shown below.

(6) $$D_{k,l}^{\left(x\right)}=\{\begin{array}{c} A\;\;if\;B_{k,l}^{\left(0\right)}\cap B_{k,l}^{\left(x\right)}=0\cap 0\\ C\;\;if\;B_{k,l}^{\left(0\right)}\cap B_{k,l}^{\left(x\right)}=0\cap 1\;\\ T\;\;if\;B_{k,l}^{\left(0\right)}\cap B_{k,l}^{\left(x\right)}=1\cap 0\;\\ G\;\;if\;B_{k,l}^{\left(0\right)}\cap B_{k,l}^{\left(x\right)}=1\cap 1 \end{array}$$

(7) $$D_{k,l}^{\left(x\right)}=\{\begin{array}{c} A\;\;if\;B_{k,l}^{\left(0\right)}\cap B_{k,l}^{\left(x\right)}=0\cap 0\\ C\;\;if\;B_{k,l}^{\left(0\right)}\cap B_{k,l}^{\left(x\right)}=0\cap 1\;\\ G\;\;if\;B_{k,l}^{\left(0\right)}\cap B_{k,l}^{\left(x\right)}=1\cap 0\;\\ T\;\;if\;B_{k,l}^{\left(0\right)}\cap B_{k,l}^{\left(x\right)}=1\cap 1 \end{array}$$

In Equations (6) and (7), *x* ∈ {1, 3, 5}. Figure 6 shows an example of forming *D*^(1)^ using the ACTG rule. The elements of *D*^(*x*)^ are synthesized to create mRNA. Thus, the following formulation holds.

(8) $$R_{k,l}=\left(D_{k,l}^{\left(1\right)}D_{k,l}^{\left(3\right)}D_{k,l}^{\left(3\right)}\right)$$

The elements *R_k,l_* are translated into *Prot_k,l_* ∈ {*I*, *L*, *V*, *F*, *M*, *C*, *A*, *G*, *P*, *T*, *S*, *Y*, *W*, *Q*, *N*, *H*, *E*, *D*, *K*, *R*, *X*}. The translation rules are listed in Table 3 and are based on the genetic codes shown in Table 2.

**Figure 8:**
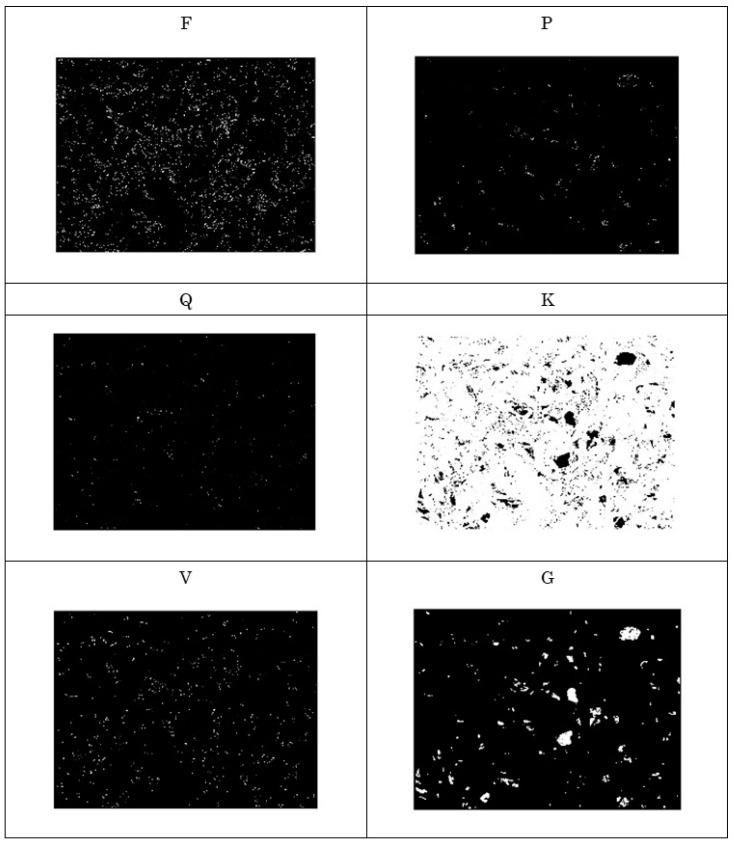
Existence of amino acids denoted as F, P, Q, K, V, and G in *Prot_k,l_* based on the ACTG rule.

**Figure 9:**
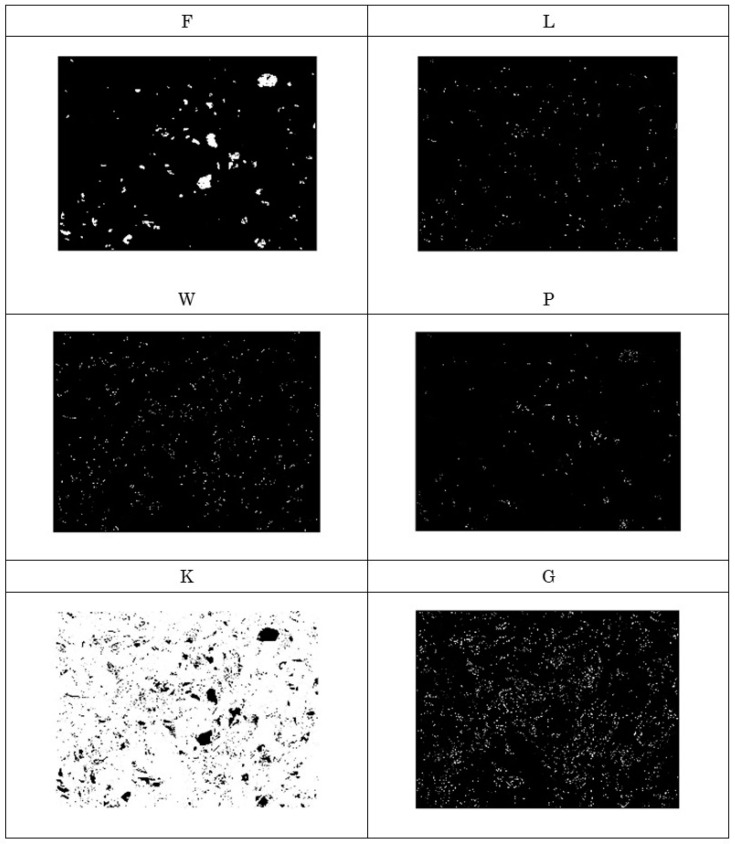
Existence of amino acids denoted as F, L, W, P, K, and G in *Prot_k,l_* based on the ACGT rule.

The existence of a specific amino acid can be shown as a white region. In this way, the protein array is transformed into a binary image. These images can be studied to solve grinding wheel surface topology problems. For example, Figure 8 shows the distributions of amino acids denoted as F, P, Q, K, V, and G in a protein where the DNA is produced using the ACTG rule. The other amino acids (not shown in Figure 8) are absent or rarely present in the protein array. As can be seen in Figure 8, the amino acid denoted as G shows relatively large and noise-free white regions distributed randomly. The corresponding binary image is thus significant for surface topography assessment. On the other hand, Figure 9 shows the distributions of amino acids denoted as F, L, W, P, K, and G in a protein array where the DNA is produced using the ACGT rule. Other amino acids (not shown in Figure 9) are absent or rarely present in the protein array. As can be seen in Figure 9, the amino acid denoted as F shows relatively large and noise-free white regions. The corresponding binary image is thus significant for surface topography assessment.

## 4. Results and Discussion

**Figure 10:**
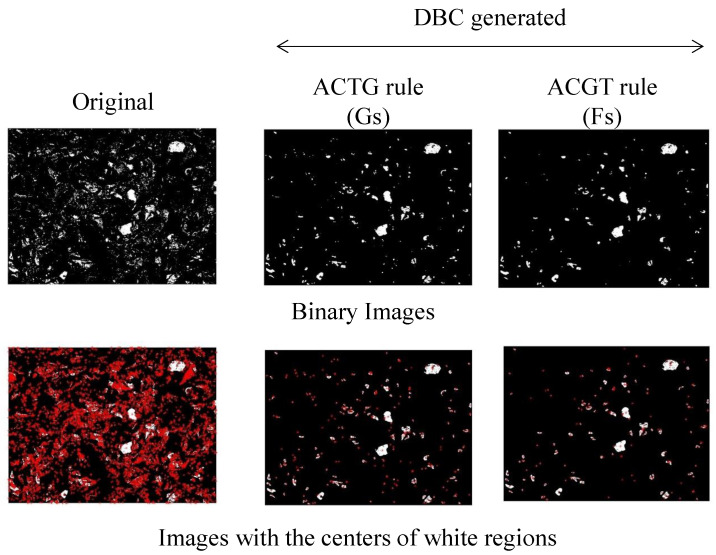
Binary images showing the efficacy of DBC.

**Figure 11:**
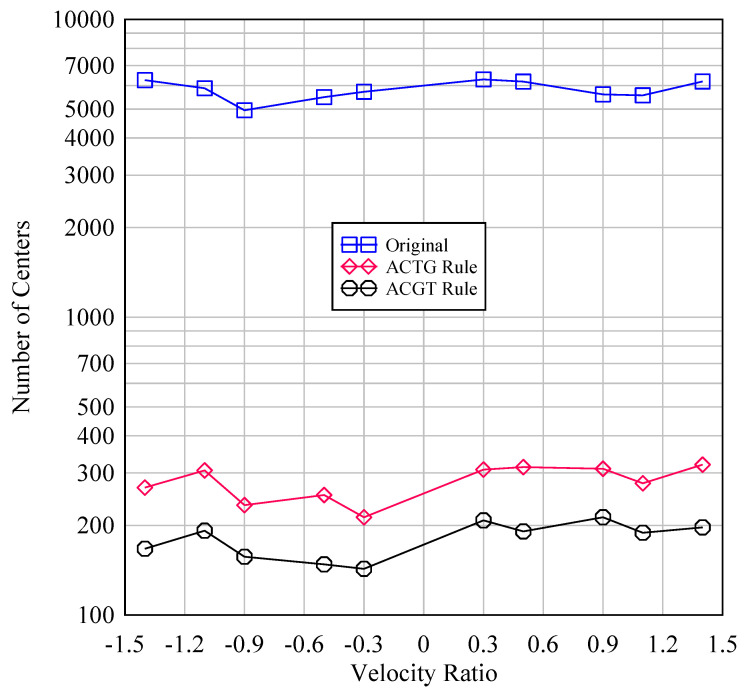
Noise reduction capacity of DBC.

The DBC method helped eliminate irrelevant areas for topography assessment. One example of this procedure is shown below. Figure 10 shows three sets of binary images, wherein each set consists of two images. The images under the script “Original” are the original images *B*^(0)^. The images under the script “ACTG rule” show the areas (white areas) where the amino acid denoted as G resided. These images were produced using the ACTG rule. On the other hand, the images under the script “ACGT rule” show the areas (white areas) where the amino acid denoted as F resided. These images were produced using the ACGT rule. The images with red dots show the centers of the white areas. As can be seen in Figure 10, the small white regions disappeared in DBC-generated binary images, whereas the large white regions remained as they were. This means that DBC helped perform Boolean transformation between useful and non-useful regions in binary images. Therefore, binary images were obtained by performing DBC according to the ACGT rule, wherein the white area represents Fs after performing DBC according to the ACGT rule. Figure 11 shows the number of centers of images, like those shown in Figure 10, for different values of velocity ratio (*VR*, see Table 1) which is a critical dressing parameter [8,9,10]. The probability of having more active abrasive grains was high when *VR* = [0.3, 0.9]. This helped optimize the dressing process more effectively. Out of the two rules, the ACGT rule helped reduce small white areas. Thus, this rule could determine the number of active abrasive grains and their distributions. 

**Figure 12:**
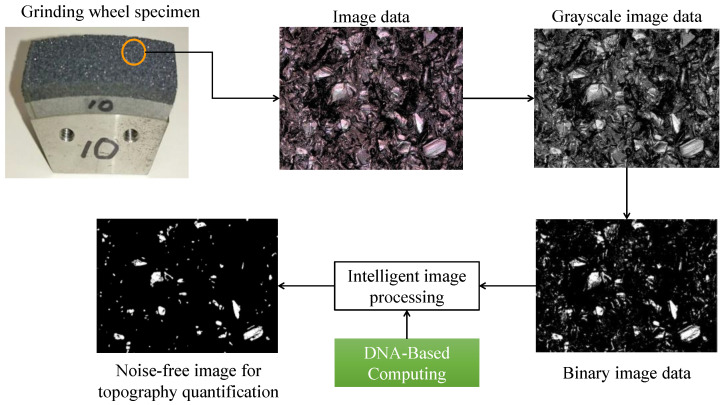
DBC-driven intelligent image processing for grinding wheel surface topography quantification.

Appendix A reports the images based on which the plot in Figure 11 is constructed. As can be seen in Figure 11, on a surface of size 706.5 μm in the circumferential direction and 530 μm in the width direction (see Table 1), there were about 7000 potential regions of possible grain residence; however, most regions were very small and did not qualify for topography determination. After performing DBC, this number was notably reduced to about 300 (which was a realistic estimate), showing that the noise which was not possible to eliminate via binary image pre-processing could be easily removed with the aid of DBC. The process is fast and can be very easily automated using commercially available image processing tools (e.g., MATLAB™ image processing toolbox). Thus, the outcomes of this study can help develop a bio-inspired, intelligent image processing system to optimize dressing operations and thereby, grinding operations, as schematically illustrated in Figure 12.

## 5. Concluding Remarks

Image processing is often employed to determine the surface topography of a dressed grinding wheel. The underlying surface topography determination process requires successful removal of regions where active abrasive grains do not reside while keeping, at the same time, the regions where active abrasive grains do reside. This results in heavy and time-consuming computation, as other authors reported. In this regard, bio-inspired DBC provides a pragmatic solution, as demonstrated in this article. It was found that on a grinding wheel surface of size 706.5 mm in the circumferential direction and 530 mm in the width direction, there were about 7000 potential regions of possible grain residence, wherein most of the regions were very small and did not qualify for topography determination. After performing DBC, this number was reduced to about 300 (which was a realistic estimate). Thus, the noise reduction ability of DBC worked well for the case of grinding wheel surface topography determination.

As enhanced “Biologicalisation in Manufacturing,” based on the convergence between biology and advanced manufacturing technology, is one of the emerging paradigms of next-generation manufacturing systems, effective, bio-inspired computing methods are greatly solicited. The presented DBC method is a contribution toward bio-inspiration endeavors in future biologicalized manufacturing systems. In this study, DBC could effortlessly eliminate unwanted image regions, represented by random distribution in binary images, while keeping, at the same time, the desired regions. Thus, DBC is particularly suitable for Boolean operations performed on binary images exhibiting complex characteristics. As a result, the DBC methodology can be used in grinding wheel surface topography determination as well as in other image processing problems relevant for intelligent manufacturing technology and systems.

## Figures and Tables

**Table 1:** Grinding wheel and other specifications.

Grinding Wheel	Vitrified CBN Grinding Wheel (BN140M100V) Diameter 140 mm, Width 10 mm
Grinding wheel rotational speed (*N*)	5000 rpm (min^−1^)
Dresser	Prismatic diamond rotary dresser Diameter 50 mm, angle 0.2 mm 120 prismatic diamond grids in the circumferential direction
Dresser rotational speed (*n*)	4200, 5600, 7000, 12,600, 15,400, and 19,600 rpm (min^−1^)
Velocity Ratio (*VR*)	±0.3, ±0.5, ±0.9, ±1.1, and ±1.4
Dressing depth of cut (*a_p_*)	2.0 µm per pass for 15 times (total depth of cut: 30 µm)
Dressing feed rate (*f*)	16.7 mm/s (0.2 mm/rev)
Coolant	WS90

**Table 2:** Genetic codes [32].

No	Amino Acids	Single-Letter Symbol of Amino Acids	Codons (In Terms of Three-Letter DNA Bases)
1	*Isoleucine*	*I*	*ATT*, *ATC*, *ATA*
2	*Leucine*	*L*	*CTT*, *CTC*, *CTA*, *CTG*, *TTA*, *TTG*
3	*Valine*	*V*	*GTT*, *GTC*, *GTA*, *GTG*
4	*Phenylalanine*	*F*	*TTT*, *TTC*
5	*Methionine*	*M*	*ATG*
6	*Cysteine*	*C*	*TGT*, *TGC*
7	*Alanine*	*A*	*GCT*, *GCC*, *GCA*, *GCG*
8	*Glycine*	*G*	*GGT*, *GGC*, *GGA*, *GGG*
9	*Proline*	*P*	*CCT*, *CCC*, *CCA*, *CCG*
10	*Threonine*	*T*	*ACT*, *ACC*, *ACA*, *ACG*
11	*Serine*	*S*	*TCT*, *TCC*, *TCA*, *TCG*, *AGT*, *AGC*
12	*Tyrosine*	*Y*	*TAT*, *TAC*
13	*Tryptophan*	*W*	*TGG*
14	*Glutamine*	*Q*	*CAA*, *CAG*
15	*Asparagine*	*N*	*AAT*, *AAC*
16	*Histidine*	*H*	*CAT*, *CAC*
17	*Glutamic acid*	*E*	*GAA*, *GAG*
18	*Aspartic acid*	*D*	*GAT*, *GAC*
19	*Lysine*	*K*	*AAA*, *AAG*
20	*Arginine*	*R*	*CGT*, *CGC*, *CGA*, *CGG*, *AGA*, *AGG*
21	*None* (*stop*)	*X ^#^*	*TAA*, *TAG*, *TGA*

^#^ Generally, the symbol “*” is used to represent stop codons. Since “*” might be confused with other symbols, the letter *X* is chosen here to represent the stop codons.

**Table 3:** Translation rules.

*Prot_k,l_*	*R_k,l_*
I	ATT, ATC, or ATA
L	CTT, CTC, CTA, CTG, TTA, or TTG
V	GTT, GTC, GTA, or GTG
F	TTT or TTC
M	ATG
C	TGT or TGC
A	GCT, GCC, GCA, or GCG
G	GGT, GGC, GGA, or GGG
P	CCT, CCC, CCA, or CCG
T	ACT, ACC, ACA, or ACG
S	TCT, TCC, TCA, TCG, AGT, or AGC
Y	TAT or TAC
W	TGG
Q	CAA or CAG
N	AAT or AAC
H	CAT or CAC
E	GAA or GAG
D	GAT or GAC
K	AAA or AAG
R	CGT, CGC, CGA, CGG, AGA, or AGG
X	TAA, TAG, or TGA

## Data Availability

Data are contained within this article.

## Appendix A. DBC-Driven Image Processing Results

This Appendix reports the datasets corresponding to Figure 11. The datasets are shown in Table A1, Table A2, Table A3, Table A4, Table A5, Table A6, Table A7, Table A8, Table A9 and Table A10 for *VR* = +0.3, +0.5, +0.9, +1.1, +1.4, −0.3, −0.5, −0.9, −1.1, and −1.4, respectively. In each table, three sets of images are shown where each set consists of two images. In each table, the left-hand-side image in the first set of images is an “Original” image, i.e., the image before performing DBC; the right-hand-side image depicts the centers of the white regions by red dots on the left-hand side image. The left-hand-side image in the second set of images shows the ACTG rule generated image; the right-hand-side image depicts the centers of the white regions by red dots on the left-hand side image. The left-hand-side image in the last set of images shows the ACGT rule generated image; the right-hand-side image depicts the centers of the white regions by red dots on the left-hand-side image. The numbers of pixels and centers of the corresponding images are also listed in Table A1, Table A2, Table A3, Table A4, Table A5, Table A6, Table A7, Table A8, Table A9 and Table A10.

**Table A1:** Images and associated pixels and number of centers corresponding to *VR* = +0.3.

*VR* +0.3	
Original	

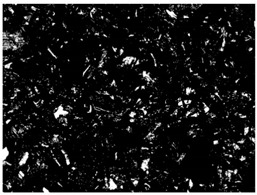
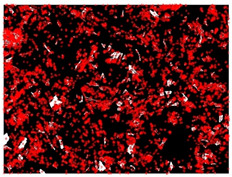
	
Pixels: 221,678	Number of Centers: 6291
ACTG Rule	

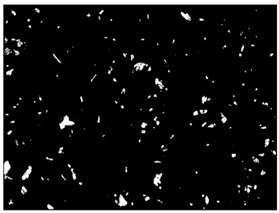
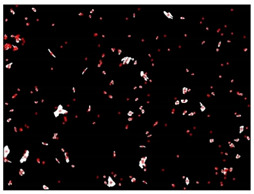
	
Pixels: 89,441	Number of Centers: 308
ACGT RULE	

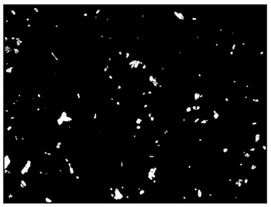
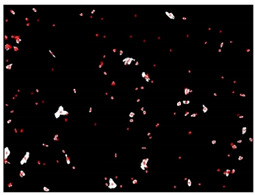
	
Pixels: 71,525	Number of Centers: 208

**Table A2:** Images and associated pixels and number of centers corresponding to *VR* = +0.5.

*VR* +0.5	
Original	

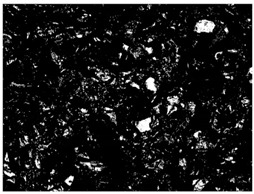
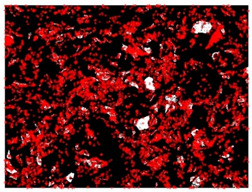
	
Pixels: 234,204	Number of Centers: 6186
ACTG RULE	

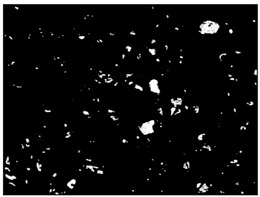
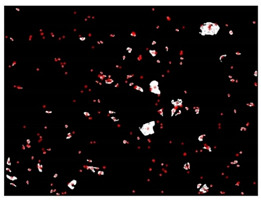
	
Pixels: 95,704	Number of Centers: 314
ACGT RULE	

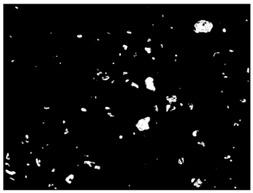
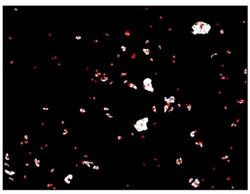
	
Pixels: 78,923	Number of Centers: 191

**Table A3:** Images and associated pixels and number of centers corresponding to *VR* = +0.9.

*VR* +0.9	
Original	

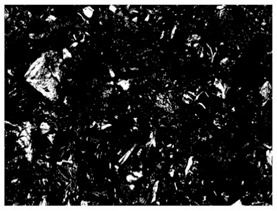
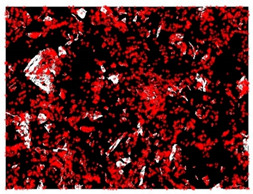
	
Pixels: 271,502	Number of Centers: 5603
ACTG RULE	

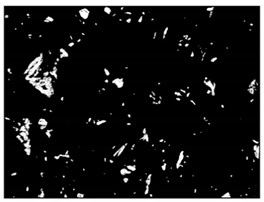
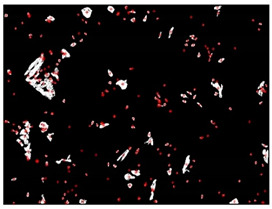
	
Pixels: 143,299	Number of Centers: 310
ACGT RULE	

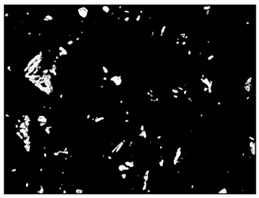
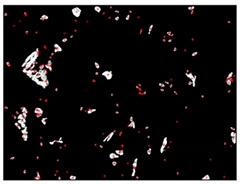
	
Pixels: 125,277	Number of Centers: 213

**Table A4:** Images and associated pixels and number of centers corresponding to *VR* = +1.1.

*VR* +1.1	
Original	

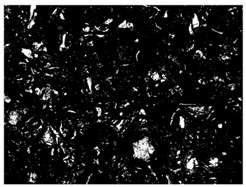
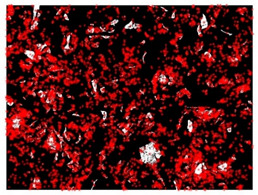
	
Pixels: 233,675	Number of Centers: 5561
ACTG RULE	

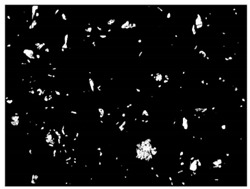
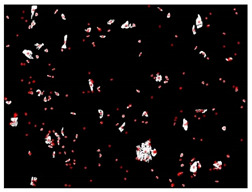
	
Pixels: 111,833	Number of Centers: 277
ACGT RULE	

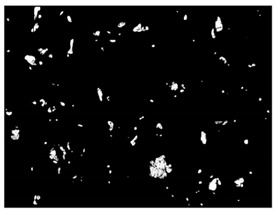
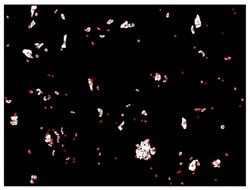
	
Pixels: 95,118	Number of Centers: 189

**Table A5:** Images and associated pixels and number of centers corresponding to *VR* = +1.4.

*VR* +1.4	
Original	

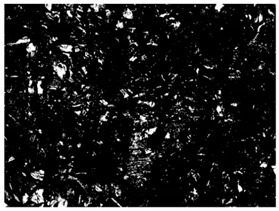
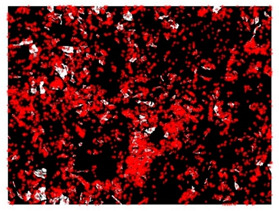
	
Pixels: 230,005	Number of Centers: 6192
ACTG RULE	

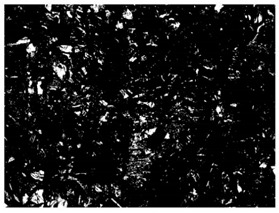
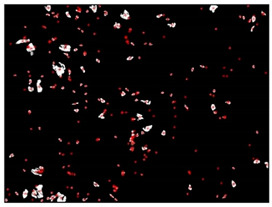
	
Pixels: 98,949	Number of Centers: 320
ACGT RULE	

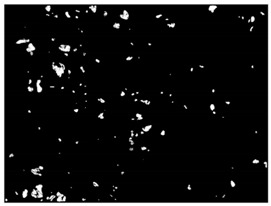
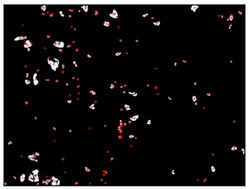
	
Pixels: 80,943	Number of Centers: 197

**Table A6:** Images and associated pixels and number of centers corresponding to *VR* = −0.3.

*VR* −0.3	
Original	

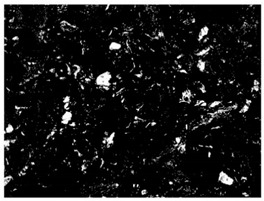
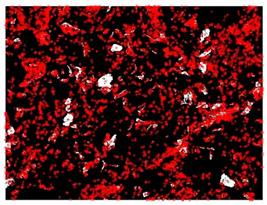
	
Pixels: 203,794	Number of Centers: 5717
ACTG Rule Generated	

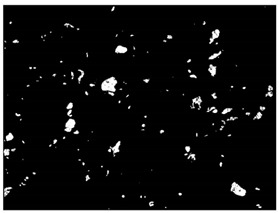
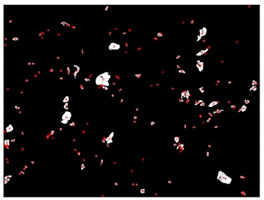
	
Pixels: 91,432	Number of Centers: 213
ACGT Rule Generated	

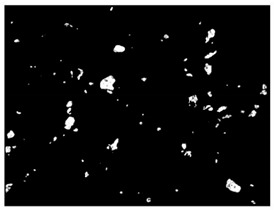
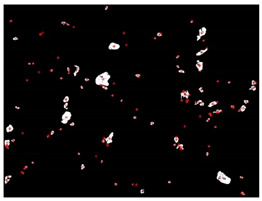
	
Pixels: 78,763	Number of Centers: 143

**Table A7:** Images and associated pixels and number of centers corresponding to *VR* = −0.5.

*VR* −0.5	
Original	

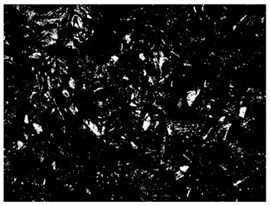
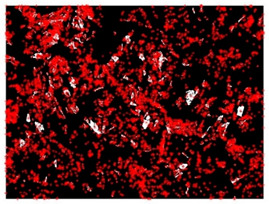
	
Pixels: 193,887	Number of Centers: 5487
ACTG Rule Generated	

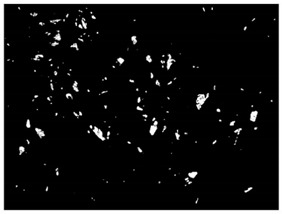
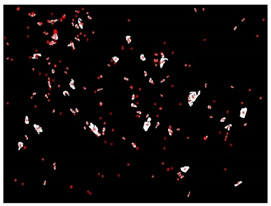
	
Pixels: 76,038	Number of Centers: 253
ACGT Rule Generated	

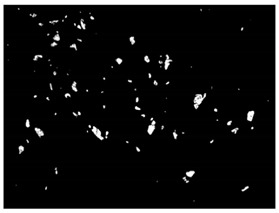
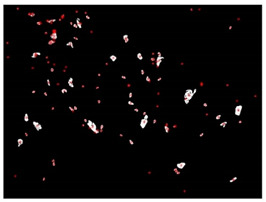
	
Pixels: 61,240	Number of Centers: 148

**Table A8:** Images and associated pixels and number of centers corresponding to *VR* = −0.9.

*VR* −0.9	
Original	

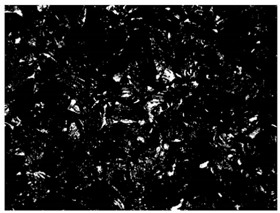
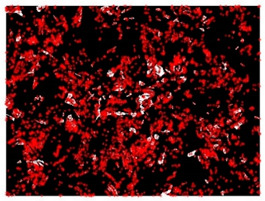
	
Pixels: 178,438	Number of Centers: 4955
ACTG Rule Generated	

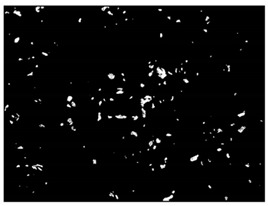
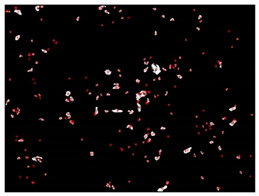
	
Pixels: 69,037	Number of Centers: 234
ACGT Rule Generated	

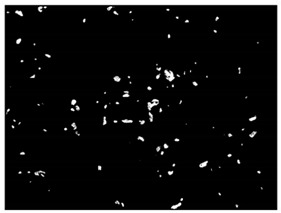
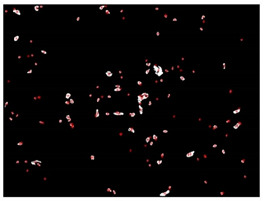
	
Pixels: 54,337	Number of Centers: 157

**Table A9:** Images and associated pixels and number of centers corresponding to *VR* = −1.1.

*VR* −1.1	
Original	

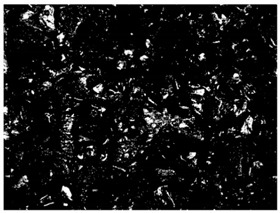
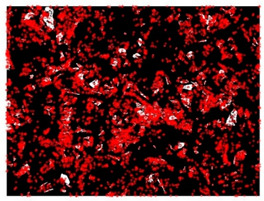
	
Pixels: 211,118	Number of Centers: 5876
ACTG Rule Generated	

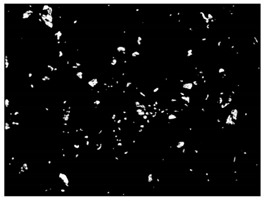
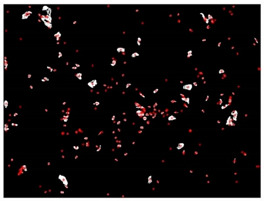
	
Pixels: 83,910	Number of Centers: 306
ACGT Rule Generated	

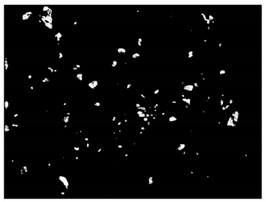
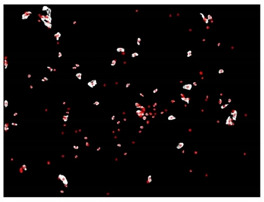
	
Pixels: 66,292	Number of Centers: 192

**Table A10:** Images and associated pixels and number of centers corresponding to *VR* = −1.4.

*VR* −1.4	
Original	

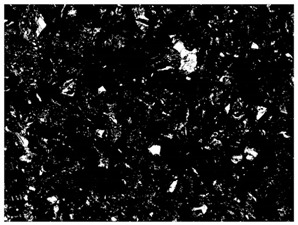
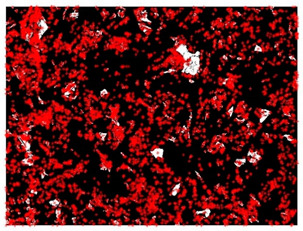
	
Pixels: 215,721	Number of Centers: 6256
ACTG Rule Generated	

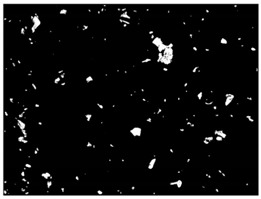
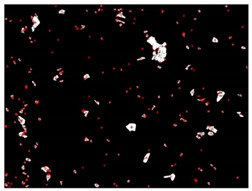
	
Pixels: 92,682	Number of Centers: 268
ACGT Rule Generated	

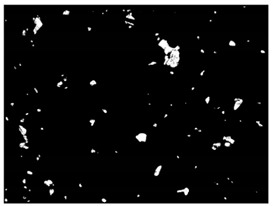
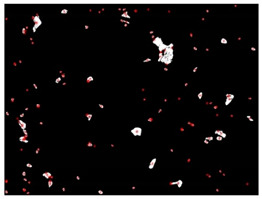
	
Pixels: 77,676	Number of Centers: 167
